# Concerning Operations for Otorrhœa

**Published:** 1907-11-09

**Authors:** 


### Otology.

### CONCERNING OPERATIONS FOR OTORRHŒA.

### II. STEELE'S ORIGINAL, AND THE RADICAL MASTOID, OPERATIONS.

(*Concluded from page 90.*)

*Stage* 4.—The opening of the attic. Stache's guard is passed from the antrum forwards through the aditus to the attic and down into the tympanum. This protects the external semi-circular canal and the facial nerve, while the deepest part of the postero-superior wall of the auditory canal is chiselled away. After the removal of the bridge of bone which forms the upper margin of the annulus tympanicus, the attic is more fully opened by chipping off the lamella which overhangs the deep part of the meatal canal superiorly.

*Stage* 5.—The removal of the remains of the ossicles and tympanic membrane. The incus and malleus are dislodged and removed, together with the remains of the tympanic membrane, with a small sharp oval curette. These parts should be carefully identified, and may be preserved for further examination if necessary. They are often imbedded in granulations or cholesteatomatous material.

*Stage* 6.—Examination of the labyrinthine windows, facial aqueduct, and exteranl semi-circular canal. To obtain a view of the stapedial fossa, the fossula rotunda, and posterior part of the tympanic floor, the middle of the floor of the meatal canal may have to be removed, though as a routine this should be avoided, owing to the extensive granulations and delayed healing consequent thereon. The facial spicule should be chipped off with a small flat chisel from the posterior margin of the tympanic ring. It is during this stage of the operation that the facial nerve is especially liable to injury, for the protector at this stage is useless. The greatest caution is necessary to avoid such a disaster. The patient's face should be constantly watched by the anæsthetist for a spasmodic twitch at the angle of the eye or the angle of the mouth. If possible, the anterior crus of the stapes should be identified. If obscured by granulation these should be removed cautiously by curetting downwards and forwards, lest the stapes be displaced and the vestibule infected. The delicate crura are liable to be broken, and no harm will result so long as the footplate be not disturbed. To ascertain the presence and state of the footplate of the stapes, a fine bent probe is passed towards the fenestra ovalis with an exceedingly light hand. In order to effect a more complete view of the fenestra ovalis, it may be necessary to remove the anterior meatal wall or retract the parotid forwards.

The fossula rotunda should be cleared of granulations, but the membrane closing the round window, and which lies in a plane at right angles to the view of the operator, should not be explored. The state of the Fallopian aqueduct and of the external semi-circular canal will have been already observed.

*Stage* 7.—The obliteration of the Eustachian tube. All granulations in the tympanum at the entrance to the Eustachian tube must be thoroughly curetted away. The occlusion of the lumen of the tube is, according to the best opinions, essential for ultimate complete healing of the operation cavity.

In spite of careful curetting, the lumen is not always obliterated, granulations persist in the tympanum, and the cavity continues to be moist. Frequent relapses of otorrhœa occur, and though some surgeons lay stress on this behaviour of the tympanic part of the wound cavity than do others, the condition is eminently satisfactory. Further progress to ensure always a dry cavity is needed. The writer practises destruction of the Eustachian mucosa by pure carbolic acid applied on small swabs, or chromic acid, or the galvano-cautery, according to circumstances. In curetting the tympanum the proximity of the internal carotid artery to the cochlea and inner tympanic wall anteriorly, and of the jugular bulb to the floor and inner wall posteriorly, should be borne in mind. The writer has in his possession a temporal bone in which the jugular bulb is separated from the middle ear, not by bone, but by fibrous tissue and mucosa only.

*Stage* 8.—The plastic operation. The object of this procedure is to secure drainage for the wound cavity through the membranous external auditory canal, so that the post-auricular wound can be closed by suture, as pointed out by Stache in 1897, and he suggested a particular device which has, however, in the writer's opinion, been improved by others, and especially by Ballance. The inferior wall of the membranous external auditory canal is incised, according to Ballance's method, with a narrow-bladed knife, passed into the meatus edge downwards. The incision is carried out into the concha, and is then continued upwards across the concha with a semi-circular curve concave forwards, and terminates at the crus of the helix, in the upper margin of the meatal outlet. Exactly how far it is desirable for the incision to sweep into the concha depends upon the size and shape of the mastoid cavity which it has been necessary to make. As a rule the incision should only extend about $\frac{1}{8}$ or $\frac{1}{4}$ of an inch into the concha from the concho-meatal margin. The tip of the little finger of the operator can now be passed into the enlarged meatus, and the posterior membranous wall is displaced backwards and upwards to the roof of the mastoid cavity. The fibro-cartilaginous tissue in the flap is dissected off from the cuticular portion, which will then be found to lie easily against the roof of the cavity without sutures. The position of the flap is maintained by filling the wound with narrow ribbon gauze, passed through a short rubber tube of ample diameter, large enough to fit the new meatal outlet.

*Stage* 9.—The closure of the wound and provision of drainage. The post-auricular wound should now be sutured with interrupted fishing gut or catgut sutures. If the mastoid cavity is large and extends to the apex of the process, a rubber tube is placed in the lower end of the wound, but not otherwise. The drainage of the greater part of the cavity has been effected according to the method described under the last stage.

*Stage* 10.—The after-treatment (local). The dry dressing can, as a rule, be left undisturbed until the fourth day, except in acute cases. Earlier dressing is indicated if there is pain or discomfort, usually on the third day; or if there is pyrexia, or if the dressing acquire an unpleasant odour, even without pain or pyrexia. Instead of remaining sweet throughout, mastoid cavities are liable to infection of proteus vulgaris and unpleasant odour-producing organisms. An adherent dressing can be removed without causing discomfort with the aid of hydrogen peroxide, 20 volumes per cent. The dressing is rapidly permeated, softened, and easily detached. The peroxide solution should be fresh. The tube if removed should be replaced for a day or two longer, and then the meatal orifice having been established, the tube may be discarded—the cavity filled with ribbon gauze daily after syringing. The dressings should be conducted with a good light thrown into the cavity, and the attention should be especially directed to the position of the meatal flap, as well as to any debris which it may be possible to remove, and so aid the healing process. In packing the cavity the tympanic region should be first filled, and then the deepest part of the mastoid in the original position of the antrum.

The stitches can be removed on the fifth day, when the patient may get up. In warm weather he may be allowed out on the seventh or eighth day. The outside dressings can be dispensed with after the second week, as a rule. Throughout convalescence the epidermisation of the cavity should be carefully watched. Any unduly prominent granulations at the growing margins of cutaneous epithelium should be kept under control by appropriate pressure with the gauze packing, or may be destroyed with pure chromic acid. Careful personal inspection is essential for uniform success.

Complete epithelialisation generally occupies about six weeks, although a small well-drained cavity in a generally healthy patient is not infrequently dry in four weeks.

Skin-grafting is not essential to secure healing, although in unusally large granulating cavities grafts certainly hasten the process.

